# Prevalence of Nutrition and Health-Related Claims on Pre-Packaged Foods: A Five-Country Study in Europe

**DOI:** 10.3390/nu8030137

**Published:** 2016-03-03

**Authors:** Sophie Hieke, Nera Kuljanic, Igor Pravst, Krista Miklavec, Asha Kaur, Kerry A. Brown, Bernadette M. Egan, Katja Pfeifer, Azucena Gracia, Mike Rayner

**Affiliations:** 1 European Food Information Council, rue Paul-Emile Janson 6, 1000 Brussels, Belgium; nera.kuljanic@gmail.com; 2 Nutrition Institute, Tržaška cesta 40, 1000 Ljubljana, Slovenia; igor.pravst@nutris.org (I.P.); krista.miklavec@nutris.org (K.M.); 3 Biotechnical Faculty, University of Ljubljana, Jamnikarjeva 101, 1000 Ljubljana, Slovenia; 4 British Heart Foundation Centre on Population Approaches for Non-Communicable Disease Prevention, University of Oxford, Oxford OX3 7LF, UK; asha.kaur@dph.ox.ac.uk (A.K.); mike.rayner@dph.ox.ac.uk (M.R.); 5 Food, Consumer Behaviour and Health Research Centre, University of Surrey, Guildford GU2 7XH, UK; kerry.brown@surrey.ac.uk (K.A.B.); m.egan@surrey.ac.uk (B.M.E.); 6 Institute for Consumer and Behavioural Research, Saarland University, 66123 Saarbrücken, Germany; k.pfeifer1@gmx.net; 7 Agrifood Research and Technology Centre of Aragon, 50059 Zaragoza, Spain; agracia@aragon.es

**Keywords:** nutrition claims, health claims, health symbols, food supply, monitoring

## Abstract

This study is part of the research undertaken in the EU funded project CLYMBOL (“Role of health-related CLaims and sYMBOLs in consumer behaviour”). The first phase of this project consisted of mapping the prevalence of symbolic and non-symbolic nutrition and health-related claims (NHC) on foods and non-alcoholic beverages in five European countries. Pre-packaged foods and drinks were sampled based on a standardized sampling protocol, using store lists or a store floor plan. Data collection took place across five countries, in three types of stores. A total of 2034 foods and drinks were sampled and packaging information was analyzed. At least one claim was identified for 26% (95% CI (24.0%–27.9%)) of all foods and drinks sampled. Six percent of these claims were symbolic. The majority of the claims were nutrition claims (64%), followed by health claims (29%) and health-related ingredient claims (6%). The most common health claims were nutrient and other function claims (47% of all claims), followed by disease risk reduction claims (5%). Eight percent of the health claims were children’s development and health claims but these were only observed on less than 1% (0.4%–1.1%) of the foods. The category of foods for specific dietary use had the highest proportion of NHC (70% of foods carried a claim). The prevalence of symbolic and non-symbolic NHC varies across European countries and between different food categories. This study provides baseline data for policy makers and the food industry to monitor and evaluate the use of claims on food packaging.

## 1. Introduction

### 1.1. Background

The use of nutrition and health-related claims (NHC) on foods and non-alcoholic beverages (henceforth foods and drinks or just foods) is regulated in many developed countries. In the European Union (EU), regulations were harmonized in 2006 by Regulation (EC) 1924/2006 [1]. The Regulation applies to all nutrition and health claims made on food packaging. The general principle is that NHC should not be misleading and should be substantiated by generally accepted scientific data [1,2]. Since the EU Register of health claims made on food entered into force in December 2012 [3], food producers in the EU are allowed to use only authorized health claims and related general non-specific health claims. One exception is claims that are still in the process of scientific evaluation, e.g., botanicals [4].

NHC as well as their symbolic representations (examples of health symbols would be the Dutch Choices logo, the Nordic Keyhole and the Finnish Heart Symbol) may help consumers identify foods that are healthier options, but little is known about how such claims are used by consumers in real-world shopping situations. The pan-European research project “Role of health-related CLaims and sYMBOLs in consumer behaviour” (CLYMBOL) has set out to determine how nutrition and health-related claims and symbols, in their context, can affect consumer understanding, purchase and consumption patterns (for an overview of the project see [5]).

As a starting point to the project, the survey reported here aimed to understand what nutrition and health-related claims and symbols consumers are exposed to, *i.e.*, on which food categories such claims and symbols are most common, which types of claims and symbols are used and to which nutrients and health relationships they refer. While some studies on the prevalence of NHC have been conducted in countries where such claims are relatively well regulated, particularly in the US [6,7], Canada [8,9], Australia [10,11,12,13] and New Zealand [14], studies on the European market are scarce and to date no cross-country prevalence study for Europe exists.

In 2009, as part of the EU-funded project FLABEL [15], a study investigated the prevalence of nutrition information, including health claims, for five food categories across all EU Member States plus Turkey [16]. Significant country-to-country differences were reported regarding the prevalence of nutrition and health claims. However, the study did not analyze the different types of claims found. More in-depth analyses are available for the UK [17], Ireland [18] and Slovenia [19]. However, all of these surveys were completed before the EU Register of health claims came into effect in 2012. Some other studies are also available in which the authors selectively focused on specific food categories, for example dairy foods [20] and breakfast cereals [21]. Lastly, the prevalence of nutrition and health claims was also recently investigated in Serbia, a candidate country for EU membership [22].

While monitoring the food supply is an important public health issue, the number of foods available and the diversity of retail environments make this challenging. A global initiative that addresses the harmonization of such data collection is the INFORMAS initiative (International Network for Food and Obesity/Non-Communicable Diseases Research, Monitoring and Action Support). This network has proposed a step-wise approach to surveying food labeling depending on resources, standardized methods for the sampling of retail outlets and foods and priorities for the labeling and related information to be collected [22]. While the selection of all foods within all food categories might be considered ideal [22], a huge volume of data is generated when studies attempt to be entirely comprehensive [19]. Therefore, a sampling approach to the selection of foods is needed particularly for studies investigating different countries. The present study describes a novel approach on how to sample a select number of foods across various countries, using the food categorization scheme [23] that has been adopted by the Global Food Monitoring Group for future monitoring of the food supply [24].

### 1.2. Research Questions

The aim of this study was to investigate the prevalence of nutrition and health claims as found on foods and drinks across five different European countries. The following research questions were identified:

- What proportion of pre-packaged foods and drinks available in-store in the five countries carry NHC?
- What proportion of these claims is symbolic?
- What types of NHC can be found on pre-packaged foods and drinks?
- To which nutrients or other food components do NHC refer?
- To which health-relationships do health claims refer?
- Which types of foods and drinks carry NHC?

## 2. Methods and Materials

Labeling data were collected from pre-packaged foods and drinks sampled in five EU countries: the UK, the Netherlands, Germany, Slovenia and Spain. The selection of countries aimed at a geographical spread across Europe. Three types of stores were selected in each country (with a total of 15 stores overall), with the aim of covering a range of different retail outlets, in order to map consumer exposure to food and drink products across a variety of shopping places: a large supermarket/national retailer, a discounter and a neighborhood store. Differences in the penetration of various food labeling information in different retail outlets were reported recently [19], confirming the rationale of the approach used in this study. Stores were selected based on the accessibility of their network within the respective country, as well as comparable store characteristics (e.g., store size). A food or drink (henceforth only referred to as “food”) was defined as a single item available for sale in the selected store. This definition meant that the same food in different sized packaging could be included in the survey, on the basis that the packaging for the same food in different sized packages may carry different health-related claims. Within-country, exact duplicates, however, were removed from the database.

Only pre-packaged foods were considered. The EU Regulation on the provision of food information to consumers (EC) 1169/2011 defines a pre-packaged foodstuff as “any single item for presentation as such to the ultimate consumer and to mass caterers, consisting of a foodstuff and the packaging into which it was put before being offered for sale, whether such packaging encloses the foodstuff completely or only partially, but in any case in such a way that the contents cannot be altered without opening or changing the packaging” [25].

### 2.1. Data Collection

In each of the five countries, approximately 400 foods were sampled *i.e.*, purchased using a randomized sampling approach. A power calculation was conducted to estimate the sample size required. An assumed 50% prevalence rate for NHC was selected in order to ensure an adequate sample size, for the detection of NHC prevalence with 5% precision. The sample size needed for an estimation of prevalence is at maximum when the measured prevalence is 50%. Hence, after adjustment for a finite population and assuming a prevalence rate of 50% for NHC, 400 foods for each country would produce confidence intervals of ±5%, sufficient for distinguishing a 10% difference in prevalence between countries.

In each of the five countries approximately 250 foods were sampled from the supermarket/national retailer, 75 foods were sampled from the discounter and 75 from the neighborhood store. Details of the whole sample are provided in Table 1.

Retailer’s store/stock lists were converted to Microsoft Excel spreadsheets, if not already in that format. The following types of product were then excluded: (a) non-food items; (b) food supplements; (c) alcoholic drinks; and (d) unpackaged foods. The remaining foods were assigned an ID number and the appropriately sized sample randomly selected using the RAND function in Excel. The sample of foods was then purchased.

For four of the stores, a store/stock list could not be obtained. In these cases a floor plan was created which mapped the layout of the store (including the location of promotional stands or other non-aisle displays). Each section/aisle was then assigned a number and the number of foods in each section/aisle was estimated using a tally counter. The total number of foods in the store was then estimated and each food location assigned an ID number. Again, an appropriately sized list of food locations was randomly generated using the RAND function in Excel. The researchers then returned to the store and purchased the foods using the list of locations.

Exclusion of products also occurred post sampling. Post-sampling exclusion occurred for unpackaged products selected inadvertently. Foods were selected and purchased in the same time frame (July–August 2013) for all countries. For perishable foods, the packaging was removed. Photographs were taken of all sides of packages carrying at least one claim.

Two pilot studies were carried out: one in Germany at a large supermarket and the other in the UK at a neighborhood store. Feedback from the pilots resulted only in minor changes to the final protocol for data collection.

### 2.2. Data Extraction

Labeling data were taken from packaging and entered into a database. All NHC were identified and characteristics of the claims were recorded, *i.e.*, whether the claim was worded, pictorial or a combination (both worded and pictorial) and whether it qualified as a symbolic claim. Worded claims were recorded verbatim and translated into English. Pictorial claims (or claims which were a combination of words and picture(s)) were briefly described. The type of health symbol was noted.

Other labeling and food composition data were also collected (e.g., whether the package had front-of-pack nutrition labeling and information about nutrient content from the nutrient declaration).

Additional characteristics of the NHC recorded included their position on pack and the number of times they appeared on one packet. For health claims, it was also noted whether it was a specific health claim, meaning whether a specified nutrient, other substance or health-related ingredient was stated to have a specific health effect, or a non-specific health claim.

### 2.3. Analysis

Once identified, claims were classified into nutrition claims (including nutrient content claims and nutrient comparative claim), health-related ingredient claims and health claims (including reduction of disease risk claims, nutrient and other function claims, general health claims and children’s development and health claims).

**Figure 1:**
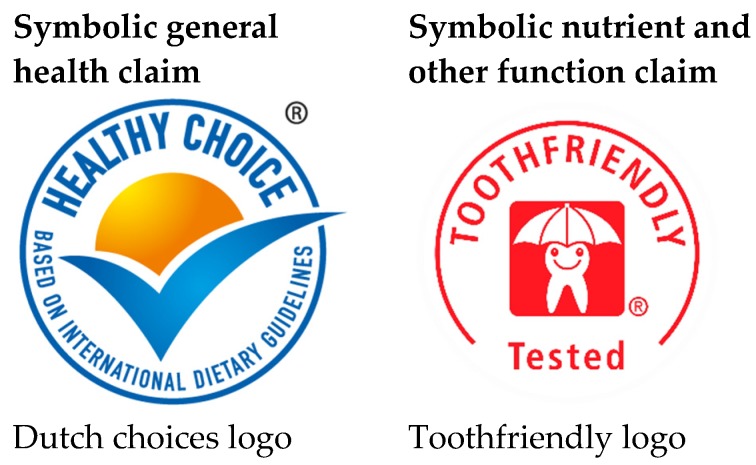
Examples of symbolic claims.

All claims were categorized as being either symbolic or non-symbolic. A symbolic claim was defined as a health-related claim (whether a nutrition, health-related ingredient or health claim) that was pictorial or combined words and pictures and for which the criteria for use of the symbol have been published. An example of a symbolic general health claim is the Dutch “Choices” logo [26]. An example of a symbolic nutrient and other function claims is the “Toothfriendly” logo [27]. see Figure 1

Color-coded nutrition labels (e.g., traffic light or % GDA labeling) did not qualify as a symbolic claim because they present nutritional information but do not suggest a health relationship [28]. All NHC definitions are consistent with EU Regulation:

1. A nutrition claim was defined as “any claim which states, suggests or implies that a food has particular beneficial nutritional properties due to (a) the energy (calorific value) it (i) provides; (ii) provides at a reduced or increased rate; or (iii) does not provide; and/or (b) the nutrients or other substances it (i) contains; (ii) contains in reduced or increased proportions; or (iii) does not contain” [1].
2. Nutrition claims were classified as either a nutrient content claim when it “describes the level of a nutrient contained in a food (or its energy value)” (e.g., “high in fibre” and “low fat”) or a nutrient comparative claim “when it compares the composition of the food in question with the composition of other foods” (e.g., “higher in fibre” and “reduced sugar”) [1].
3. A health-related ingredient claim was defined as a claim communicating the presence of an ingredient(s) which is not a nutrient or other substance as defined in the EU Regulation [1] but which implies health benefits. In most cases, these claims related to the content of ingredients that are considered as a healthy (e.g., “Contains one of your five a day”) or at least a healthier alternative (e.g., “Sweetened only with brown sugar”).
4. A health claim was defined as “any claim that states, suggests or implies that a relationship exists between a food category, a food or one of its constituents and health” [1].
5. A general health claim (covered by Article 10(3) of the EU Regulation [1]) was defined as a claim referring to benefits for general health or well-being. A typical example of such a claim is “Good for your health” or “Healthier choice (within this product group)”.
6. A nutrient and other function claim (as covered by Article 13 of the EU Regulation [1]) was defined as a health claim that describes or refers to one of the following: (a) the role of a nutrient or other substance in growth, development and the functions of the body; (b) psychological and behavioral functions; or (c) slimming or weight-control or a reduction in the sense of hunger or an increase in the sense of satiety or a reduction of the available energy from the diet. Typical examples of such a claim are “Calcium builds strong teeth” and “Fibre helps maintain a healthy digestive system”.
7. A reduction of disease risk claim (covered by Article 14.1(a) of the EU Regulation [1]) was defined as a claim communicating that the consumption of a food category, a food or one of its constituents significantly reduces a risk factor in the development of a human disease. Typical examples of such a claim are “Plant sterols reduce blood cholesterol. High cholesterol is a risk factor in the development of coronary heart disease” and “Reduces the risk factor for development of dental caries”.
8. A children’s development and health claim (covered by Article 14.1(b) of the EU Regulation [1]) was defined as a health claim where children’s development and/or health was specifically mentioned. Typical examples of such a claim are “Calcium is needed for normal growth and development of bones in children” and “For your baby’s safe and balanced diet”. Claims where children were not specifically mentioned, even if these claims were found on foods intended solely for use by children, were not considered children’s development and health claims.

Claims such as “natural”, “organic” and “halal” were not considered NHCs on the basis that they refer to the method of the production rather than the content. Similarly, information on the presence of additives, preservatives, flavorings, *etc.* was not considered a health-related claim. Allergy advice (e.g., “contains nuts”) was not considered to be an NHC nor were references to the presence of a food or food group in the product where there was no clear relationship to its health benefits, e.g., references to milk content. Claims relating to the endorsement of a health-related organization were also not included, e.g., “[Product] supported by the [health association]”.

## 3. Results

Where confidence intervals are provided in tables, they will not be reported in the text. Results are discussed by country, claim type and product category.

### 3.1. What Proportion of Pre-Packaged Foods and Drinks Available In-Store in the Five Countries Carry NHC?

Twenty-six percent of foods carried at least one nutrition, health-related ingredient or health claim (including symbolic versions of such claims) (Table 2). There were almost twice as many foods carrying nutrition claims as there were foods carrying health claims (21% and 11% respectively). Just 4% of foods carried a health-related ingredient claim. General health claims appeared on 7% of the foods. Only 5% of foods carried a nutrient and other function claim. Just 0.6% of foods carried a reduction of disease risk claim and 0.7% of foods carried children’s development and health claims. Products could carry more than one claim.

Thirty percent of foods sampled in the UK carried a nutrition claim, followed by Spain (23%), Slovenia (19%), the Netherlands (17%) and Germany (16%). There was less variation across countries in the number of foods carrying health claims; the highest prevalence was observed in the Netherlands (14%) and the lowest in Spain (7%). The foods sampled in the Netherlands had more instances of general health claims (12%), compared to 4%–6% for the remaining countries. The highest proportion of foods carrying a nutrient and other function claim was found in Slovenia (9%), followed by the UK (7%), Germany (5%), Spain and the Netherlands (both 3%). Overall, the prevalence of reduction of disease risk claims was very low across all countries; it was highest in the UK (1%) and lowest in the Netherlands (0.2%). Similarly, the prevalence of children’s development and health claims ranged from 0% (in the Netherlands) to 1.3% (in Germany).

Foods carrying claims tend to carry multiple claims; the average number of NHC per product carrying any claim across the five countries was 2.6 (Table 3). The highest number of claims seen on a product was 17, observed on two baby food products in Spain and Germany. The mean number of nutrition claims per product carrying any claim was slightly higher than the mean number of health claims (2.0 and 1.9, respectively). A Kruskal-Wallis test was used to determine if there were any significant country differences in the number of nutrition claims, health claims or any claims (NHC) of foods that carry such claims. There was a significant country difference in the number of health claims, but the country differences in the number of nutrition claims or NHC claims overall were not statistically significant. The highest number of health claims observed on a single product was 15, found on a baby food, and for nutrition claims, 13 claims found on a confectionery product, both in Germany.

### 3.2. What Proportion of These Claims is Symbolic?

Substantial differences were observed in the prevalence of symbolic claims between the countries (Table 2). The highest prevalence was found in the Netherlands (12%, (9%–14%)), followed by Spain (4%, (2%–6%)), Slovenia (2%, (1.0%–3.0%)), the UK (1%, (0%–2%)), and Germany (0.3%, (0%–1%)). Almost all of the general health claims in the Netherlands were symbolic—the majority of which comprised of the Choices logo. Spain had a similarly high level of symbolic general health claims, including logos of health organizations such as the Spanish Association for Pediatricians. However, it should be noted that in general the prevalence of symbolic claims was quite low; altogether only 3.88% (3%–5%) of foods were labeled with a symbolic claim.

### 3.3. What Types of NHC Can Be Found on Pre-Packaged Food and Drinks?

64% of the claims were nutrition claims of which 92% (90%–94%) were classified as nutrient content claims and just 8% (6%–10%) were nutrient comparative claims (Table 2). Health claims accounted for 29% (26%–31%) of the claims recorded. Of these, almost half (47%, (42%–52%)) were nutrient and other function claims, 39% (34%–44%) were general health claims, 8% (6%–11%) were children’s development and health claims and the remaining 5% (3%–8%) were reduction of risk claims. The remaining claims fell into the category of health-related ingredient claims (8%, (6%–9%)).

The proportion of nutrition claims was highest in Spain (74% of all claims were nutrition claims), followed by the Netherlands (64%), the UK (62%), Slovenia (61%), and Germany (55%). The proportion of health claims showed slightly less variation across the five countries: it was highest in Slovenia and Germany (both 37%), followed by the Netherlands (31%), Spain (24%) and the UK (21%).

Only 14% (12%–15%) of the NHC were nutrient and other function health claims. The highest proportion was found in Slovenia (25%, (19%–30%)) and lowest in the Netherlands (8%, (5%–11%)). Spain had the highest proportion of children’s development and health claims (6%, (3%–9%)) while none were found on foods sampled in the Netherlands. The UK had the highest proportion of disease risk claims (3%, (1%–4%)) whereas Germany had the lowest (only one such claim was found, out of a total of 224 claims identified) (Table 2).

### 3.4. To Which Nutrients or Other Food Components Do NHC Refer?

More than one third of the nutrition claims referred to vitamins and/or minerals (e.g., “Enriched with important vitamins and minerals”) (Table 4). More specifically, 22% referred to vitamins, of which vitamin C was the most common vitamin referenced (e.g., “High in vitamin C”) and 13% to minerals, of which calcium was the most common mineral (e.g., “A source of calcium”). Almost a quarter of nutrition claims (24%) referred to the fat content of a food in some way, 12% to the sugar content and 9% to fiber.

Thirty-six percent of health claims referred to an unspecified nutrient or nutrients (e.g., “Complete nutrition for optimal growth”) and 21% referred to the whole food (e.g., the claim “Cholesterol reducing” without specifying a nutrient) (Table 4). Sixteen percent of health claims referred to vitamins or minerals. Of the health claims that referred to macronutrients, references to fat were the most common.

### 3.5. To Which Health-Relationships Do Health Claims Refer?

All health claims were categorized following the International Classification of Functioning, Disability and Health (ICF) [29], in order to ensure comparability with outputs from, e.g., the World Health Organization (WHO). Functions of the digestive, metabolic and endocrine system were referred to in 40% of nutrient and other function claims (Table 5). Of these, 57% (46%–69%) referred to functions related to general metabolic functions and/or weight management functions (e.g., “Weight watchers”). Fifteen percent of nutrient and other function claims referred to functions of the cardiovascular, hematological, immunological and respiratory systems (e.g., “Zinc helps maintain a healthy immune system”), 11% referred to mental functions (e.g., “Glycemic carbohydrates contribute to the maintenance of healthy brain functions”) and 10% referred to neuro-musculoskeletal and movement-related functions (e.g., “Growing strong bones”).

### 3.6. Which Types of Foods Carry NHC?

**Figure 2:**
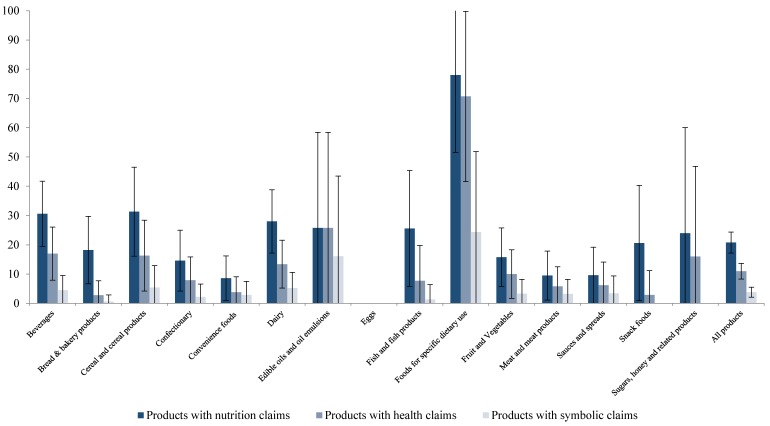
Prevalence of nutrition and health claims (including symbolic ones) by food category. (*Note*: % of foods with claim, including the confidence intervals at 95% (see also Supplementary Material Table S1)).

The prevalence of health-related claims and symbols for all countries, across all food categories, is presented in Figure 2. “Foods for specific dietary uses” had the highest proportion of nutrition, health and symbolic claims (78%, 71%, and 24%, respectively). According to the classification scheme used in this study [23], this category includes foods intended for babies and infants (e.g., milk formulas and follow-on foods) but also meal replacements (e.g., diet shakes). In this sample, only baby foods were found. This corresponds to the findings reported in Table 3 where the highest number of claims found on a single product (17) was on two baby foods in Germany and Spain. It should be noted that the category of “Foods for specific dietary uses” only represents 2% (0.01%–0.03%) of all foods sampled in this study.

Almost a third of “Cereal and cereal products” and “Beverages” carried nutrition claims, followed by “Dairy products” (28%), “Edible oils and oil emulsions” and “Fish and fish products” (both 26%) and “Sugars, honey and related products” (24%) (Figure 2). The remaining categories had 20% or less of foods carrying nutrition claims. Twenty-six percent of “Edible oils and oil emulsions” carried health claims, followed by “Beverages” (17%), “Cereal and cereal products” and “Sugars, honey and related products” (both 16%), and “Dairy products” (13%). The remaining food categories had ≤10% of foods carrying a health claim. All details can be found in the Supplementary Material (Table S1).

## 4. Discussion

The present study assesses the prevalence of NHC on packaged foods currently sold in Germany, the Netherlands, Slovenia, Spain and the UK. Overall, approximately one quarter (26%) of foods sampled in this study carried an NHC. Twice as many foods carried nutrition claims (21%) compared to health claims (11%), followed by a much smaller percentage of health-related ingredient claims (4%).

On a country-level, the overall percentage of foods with at least one claim varied from 35% in the UK to 21% in Germany. The UK also had the highest number of foods carrying a health-related ingredient claim (10%), compared to only two foods (0.5%) surveyed in Slovenia. UK health-related ingredient claims were mostly related to fruit and vegetable consumption (e.g., “One of your five a day”) or wholegrain (e.g., “With wholegrain”). Regarding health claims, country-by-country variation was lower and did not follow the same distribution order. The highest frequency of foods carrying at least one health claim (including symbolic representations) was found in the Netherlands and the lowest frequency in Spain. A closer look at the Dutch data show that the majority of health claims identified here comprised of the Choices logo, which was classified as a symbolic general health claim. All five surveyed countries have been governed by the same European food regulation, since 2007. Our results demonstrate that reasons for the reported country differences regarding the prevalence of health claims and symbols appear to be more complex than just the implementation of a supranational regulation. EU Member States differ in their history of use of health claims and symbols prior to the EU regulation, food operators in different countries may employ different marketing strategies for their products, and the use of health symbols is often linked to national organizations issuing those symbols. Furthermore, it has been reported that EU Member States take different approaches to implementing the EU regulation on a national level. This includes national legislation on the responsibilities of food authorities (e.g., inspections), as well as the general control of the food supply [30]. These differences are currently being investigated in another on-going EU funded project called REDICLAIM (www.rediclaim.eu).

It appears that when foods carry claims, they tend to carry more than one claim, which can either mean that they repeat the same claim on several parts of the package or include more than one claim. The latter is often the case for nutrition claims, *i.e.*, several nutrients are mentioned (e.g., “No sugar, low calories” and “Contains vitamin A, source of iron”). Furthermore, because health claims are commonly communicating functions of specific nutrients or other substances, such claims are commonly accompanied by related nutrition claims (e.g., “High in calcium” and “Calcium is needed for the maintenance of normal bones”).

Due to differences in methods, data collection timeframe, countries and food categories surveyed, it is difficult to compare the present findings with those of past surveys and any comparison should be undertaken with caution. Nevertheless, it is useful to note the prevalence of claims that previous studies have reported.

Of the products sampled in this study, 21% carried nutrition claims, compared to 29% that were reported in a study undertaken in the UK in 2011 [17], 37% in Slovenia in 2011 [19] and 48% in Ireland in 2007 [18]. In Serbia, a candidate country for EU membership, 7% were reported in a study undertaken in 2012 [22]. Outside Europe, similar studies reported 46% of foods carrying nutrition claims in Canada in 2013 [9] and 49% in the USA in 2010 [7]. Compared to 11% of products carrying health claims in this study, a prevalence of 15% health claims on food products was reported in the British study [17], 13% in the Slovenian study [19] and 18% in the Irish study [18]. The Serbian study only reported 6% health claims on the food products they sampled [22]. It should be noted that health claims were not regulated in Serbia at the time that the study was done. Outside the EU, 14% health claims were found on food products in a study undertaken in Australia and New Zealand [11] and 9% in the US-based study [7]. While the highest proportion of health claims in the present study comprised of general health claims (7%), only few reduction of disease risk claims (0.6%) were identified. This is in line with findings from other prevalence studies, e.g., <1% of reduction of disease risk claims in the British [17], the Irish [18], the Slovenian [19] and the Canadian study [9] and only 1% in the Australian and New Zealand study [11]. Regarding the prevalence of symbolic claims, the present study reported such claims on 4% of all food packages. The highest proportion of symbolic claims was observed in the Netherlands (12%) and the lowest in Germany (0.3%), which is in line with the results of the FLABEL study mentioned above [16]. The US study reported that 6% of food packages had symbolic claims [7]. A comparison with the Canadian study [9] is not possible due to differences in the definition of symbolic claims between that study and this.

### 4.1. Strengths

The present study is the first, to-date, to survey several countries across Europe using the same methods, with data collection having taken place after the Regulation went into effect. Aside from providing an updated overview of the prevalence of nutrition, health-related ingredient and health claims on the European market, the present study offers a novel method of data collection in which the sampling frame was rigorously defined. It is hoped that the detailed description of the methods will provide guidance for other researchers on replicating and advancing future claim prevalence studies.

Furthermore, the inclusion of five different countries and the geographical spread that this entails has allowed for European conclusions to be drawn. This study provides an example of how to monitor the prevalence of claims currently on the market, one of the tasks crucial in implementing and monitoring legislation. It is also a necessary step towards making research a useful source of information for future regulation. At the same time, differences across European countries were taken into account by using local researchers for the data extraction. Nuances in the language were more likely to be appreciated by native speakers in each country. This approach required more coordination efforts but the authors believe that this was appropriate and would be the most suitable method for future similar research.

### 4.2. Limitations

The study was powered to detect a 10% difference in the prevalence of claims by country, which had an effect on comparisons in other respects. For example, the sample size did not allow for country-by-country analysis for each food category. It also did not allow for a store-by-store analysis for each claim type. Having limited resources, the decision was either to focus on a few food categories and the number of investigated jurisdictions or to broaden the number of food categories and countries while limiting the number of foods per country. The latter option was seen as a better way to produce a snapshot of the current situation in the EU market, *i.e.*, what consumers are exposed to, across a variety of stores and food categories.

The selection of an appropriate food categorization system has posed a further challenge in this study. A variety of categorization schemes have been used in similar studies, which significantly limits the comparability of results. The selected scheme was chosen due to the fact that it has been adopted by the Global Food Monitoring Group for all future monitoring of the food supply [23,24]. Although this study is the first to use this categorization scheme for this type of labeling survey, it is the authors’ hope that this decision will contribute to the harmonization of food information reporting in future studies.

Last but not least, the cross-sectional design can be considered an additional limitation of this study. In order to examine changes in the food supply, it is recommended to repeat this study at a later point, e.g., after all transition periods of the EU Regulation have ended. A further avenue of future research could be to analyze differences in the prevalence of claims between retail (private label) products and branded products.

## 5. Conclusions

The prevalence of symbolic and non-symbolic NHC varies across European countries and between different food group categories. Nutrition and/or health claims were found on about a quarter of surveyed pre-packaged foods in five EU countries. The majority of all claims were nutrition claims, followed by health claims and health-related ingredient claims. Health claims were mostly present in the form of nutrient and other function claims, while disease risk reduction and children’s development and health claims were observed on less than 1% of the foods. The results of this study not only provide baseline data for policy makers to monitor the use of claims in food information to consumers but also offer the potential to inform future regulation. Results are also important for subsequent phases of CLYMBOL studies involving consumer understanding and use of such information in purchase and consumption behavior.

## Figures and Tables

**Table 1:** Sample overview (sampling method and number of foods).

	UK	Netherlands	Germany	Slovenia	Spain	Total
Large supermarket/ national retailer	floor plan, *n* = 248	store list, *n* = 252	store list, *n* = 248	store list, *n* = 260	store list, *n* = 251	1259 (62%)
Discounter	floor plan, *n* = 75	store list, *n* = 81	store list, *n* = 76	store list, *n* = 78	store list, *n* = 78	388 (19%)
Neighborhood store	floor plan, *n* = 75	store list, *n* = 83	floor plan, *n* = 75	store list, *n* = 78	store list, *n* = 76	387 (19%)
Total no of foods	398 (20%)	416 (20%)	399 (20%)	416 (20%)	405 (20%)	2034 (100%)

Foods were sampled from retail outlets in two ways: either using a store/stock list or failing that a floor plan.

**Table 2:** Prevalence of nutrition claims, health claims, and symbols in five European countries.

Country	Claim Type	No. of Claims	… of Which Are Symbolic	No. of Foods with a Claim	% of Foods with Claim (95% CIs)
All Countries N = 2034 Foods	**Nutrition claim**	**865**	**1**	**423**	**20.8% (19.0%–22.5%)**
	*Nutrient content claim*	*797*	**1**	*399*	*19.6% (17.8%–21.3%)*
	*Nutrient comparative claims*	*68*	**0**	*49*	*2.4% (1.7%–3.1%)*
	**Health-related ingredient claim**	**105**	**6**	**72**	**3.5% (2.7%–4.3%)**
	**Health claim**	**392**	**74**	**222**	**10.9% (9.6%–12.3%)**
	*General health claim*	*153*	*64*	*137*	*6.7% (5.6%–7.8%)*
	*Nutrient and other function claim*	*185*	*9*	*106*	*5.2% (4.2%–6.2%)*
	*Reduction of disease risk claim*	*21*	*1*	*12*	*0.6% (0.2%–0.09%)*
	*Children’s development & health claims*	*33*	*0*	*15*	*0.7% (0.4%–1.1%)*
	**Any type of claim (NHC)**	**1362**	**81**	**528**	**26.0% (24.0%–27.9%)**
UK N = 398 foods	**Nutrition claim**	**247**	**0**	**118**	**29.6 (25.1%–34.1%)**
	**Health-related ingredient claim**	**65**	**3**	**40**	**10.1% (7.1%–13.0%)**
	**Health claim**	**85**	**2**	**44**	**11.1% (8.0%–14.1%)**
	*General health claim*	*30*	*0*	*23*	*5.8% (3.5%–8.1%)*
	*Nutrient and other function claim*	*38*	*2*	*26*	*6.5% (4.1%–9.0%)*
	*Reduction of disease risk claim*	*10*	*0*	*4*	*1.0% (0.0%–0.2%)*
	*Children’s development & health claims*	*7*	*0*	*4*	*1.0% (0.0%–2.0%)*
	**Any type of claim (NHC)**	**397**	**5**	**140**	**35.2% (30.4%–40.0%)**
Nether-lands N = 416 foods	**Nutrition claim**	**154**	**0**	**70**	**16.8% (13.2%–20.4%)**
	**Health-related ingredient claim**	**12**	**0**	**12**	**2.9% (1.3%–4.5%)**
	**Health claim**	**73**	**50**	**60**	**14.4% (8.9%–15.2%)**
	*General health claim*	*52*	*49*	*50*	*12.0% (8.9%–15.2%)*
	*Nutrient and other function claim*	*19*	*1*	*12*	*2.9% (1.3%–4.5%)*
	*Reduction of disease risk claim*	*2*	*0*	*1*	*0.2% (0.0%–0.1%)*
	*Children’s development & health claims*	*0*	*0*	*0*	*0.0%*
	**Any type of claim (NHC)**	**239**	**50**	**103**	**24.8% (20.6%–29.0%)**
Germany N = 399 foods	**Nutrition claim**	**123**	**0**	**64**	**16.0% (12.4%–19.7%)**
	**Health-related ingredient claim**	**19**	**1**	**13**	**3.3% (1.5%–5.0%)**
	**Health claim**	**82**	**0**	**37**	**9.3% (6.4%–12.1%)**
	*General health claim*	*29*	*0*	*23*	*5.8% (3.5%–8.1%)*
	*Nutrient and other function claim*	*45*	*0*	*20*	*5.0% (2.9%–3.2%)*
	*Reduction of disease risk claim*	*1*	*0*	*1*	*0.3% (–0.2%–0.7%)*
	*Children’s development & health claims*	*7*	*0*	*5*	*1.3% (0.1%–0.2%)*
	**Any type of claim (NHC)**	**224**	**1**	**82**	**20.6% (16.6%–24.5%)**
Slovenia N = 416 foods	**Nutrition claim**	**144**	**0**	**78**	**18.8% (15.0%–22.5%)**
	**Health-related ingredient claim**	**3**	**1**	**2**	**0.5% (–0.2%–1.1%)**
	**Health claim**	**88**	**7**	**52**	**12.5% (0.9%–15.7%)**
	*General health claim*	*24*	*1*	*23*	*5.5% (3.3%–7.7%)*
	*Nutrient and other function claim*	*58*	*6*	*36*	*8.7% (5.9%–11.4%)*
	*Reduction of disease risk claim*	*4*	*0*	*3*	*0.7% (–0.1%–1.5%)*
	*Children’s development & health claims*	*2*	*0*	*1*	*0.2% (–0.2%–0.7%)*
	**Any type of claim (NHC)**	**235**	**8**	**103**	**24.8% (20.6%–28.9%)**
Spain N = 405	**Nutrition claim**	**196**	**1**	**93**	**23.0% (18.8%–27.1%)**
	**Health-related ingredient claim**	**6**	**1**	**5**	**1.2% (0.2%–2.3%)**
	**Health claim**	**64**	**15**	**29**	**7.2% (4.6%–9.7%)**
	*General health claim*	*18*	*14*	*18*	*4.4% (2.4%–6.5%)*
	*Nutrient and other function claim*	*25*	*0*	*12*	*3.0% (1.3%–4.6%)*
	*Reduction of disease risk claim*	*4*	*1*	*3*	*0.7% (–0.1%–1.6%)*
	*Children’s development & health claims*	*17*	*0*	*5*	*1.2% (0.2%–2.3%)*
	**Any type of claim (NHC)**	**266**	17	**100**	**24.7% (20.5%–28.9%)**

Bold is for main claims we looked at and the total claims. Italic is for the sub-categories of all these claims. Regular font size was only used for the description of the countries to the left.

**Table 3:** Number of claims per product.

	Mean Number of Nutrition Claims ^1^	Highest Number of Nutrition Claims on a Single Product	Mean Number of Health Claims ^1^	Highest Number of Health Claims on a Single Product	Mean Number of Any Claim ^1^	Highest Number of Any Claims on a Single Product
All countries	2.0	13	1.9	15	2.6	17
UK	2.1	11	1.9	5	2.8	15
Netherlands	2.2	8	1.2	6	2.3	9
Germany	1.9	13	2.2	15	2.7	17
Slovenia	1.8	8	1.7	5	2.3	11
Spain	2.1	12	2.2	11	2.7	17
*p* value	0.94		<0.01		0.52	

NOTE: per product carrying a claim.

**Table 4:** Nutrients and ingredients referred to in nutrition and health claims (all five countries).

Nutrient	Nutrition Claim (No.)s	% of All Nutrition Claims	Health Claim (No.)s	% of All Health Claims
Energy	40	5%	1	<1%
Protein	35	4%	8	2%
Carbohydrates	109	13%	14	4%
Of which sugars	100	12%	2	1%
Fat	206	24%	31	8%
Total fat	127	15%	5	1%
Saturated fat	7	1%	3	1%
Unsaturated fat	50	6%	23	6%
*Omega-3 fatty acids*	33	4%	15	4%
Fiber	74	9%	14	4%
Sodium/Salt	35	4%	0	0%
Vitamins and/or minerals	305	35%	64	16%
Vitamins and Minerals	2	<1%	3	1%
Vitamins (any)	187	22%	38	10%
*Vitamin C*	47	5%	8	2%
*Vitamin D*	15	2%	9	2%
*Vitamin E*	19	2%	5	1%
*Other specified vitamins*	55	6%	11	3%
*Unspecified vitamins*	51	6%	5	1%
Minerals (any)	116	13%	23	6%
*Calcium*	55	6%	13	3%
*Iron*	21	2%	3	1%
*Other specified minerals*	26	3%	6	2%
*Unspecified minerals*	14	2%	1	<1%
Probiotics	23	3%	2	1%
Phytosterols/stanols	6	1%	5	1%
Whole products	0	0%	84	21%
Unspecified nutrient	4	<1%	141	36%
Other nutrients	27	3%	9	2%
Ingredients that are not nutrients	1	<1%	19	5%
Herbs	0	0%	12	3%
Seeds	0	0%	3	1%
Whole grain/Whole wheat / Whole foods/Whole meal	1	<1%	2	1%
TOTAL	865	100%	392	100%

**Table 5:** Distribution of nutrient and other function claims by the International Classification of Functioning, Disability, and Health (ICF).

ICF Chapter		No. of Claims	% of Claims (95% CIs)
Mental Functions	Global psychosocial functions	4	2.2% (0.0%–4.3%)
	Energy and drive functions	8	4.4% (1.4%–7.4%)
	Sleep functions	2	1.1% (–0.4%–0.26%)
	Specific mental functions	1	0.5% (–0.5%–1.6%)
	Higher-level cognitive functions	1	0.5% (–0.5%–1.6%)
	**Total**	**20**	11.0% (6.4%–15.6%)
Sensory Functions and Pain	Seeing and related functions	1	0.5% (–0.5%–1.6%)
	**Total**	**1**	0.5% (–0.5%–1.6%)
Voice and Speech Functions	Voice functions	4	2.2% (0.0%–4.3%)
	**Total**	**4**	2.2% (0.0%–4.3%)
Functions of the cardiovascular, hematological, immunological and respiratory systems	Heart functions	9	4.9% (1.8%–8.1%)
	Blood vessel functions	1	0.5% (–0.5%–1.6%)
	Immunological system functions	16	8.8% (4.6%–12.9%)
	Functions of the respiratory system	1	0.5% (–0.5%–1.6%)
	**Total**	**27**	14.8% (9.6%–20.0%)
Functions of the digestive, metabolic and endocrine systems	Digestive functions	19	10.4% (6.0%–14.9%)
	Defecation functions	5	2.7% (0.3%–5.1%)
	Weight maintenance functions	14	7.7% (3.8%–11.6%)
	General metabolic functions	27	14.8% (9.6%–20.0%)
	Water, mineral and electrolyte balance functions	6	3.3% (0.7%–5.9%)
	Endocrine gland functions	2	1.1% (–0.4%–2.6%)
	**Total**	**73**	40.1% (32.9%–47.3%)
Genitourinary and reproductive functions	Urinary excretory functions	1	0.5% (–0.5%–1.6%)
	Sexual functions	2	1.1% (–0.4%–2.6%)
	**Total**	**3**	1.6% (–0.2%–3.5%)
Neuro-musculoskeletal and movement related functions	Functions of the joints and bones	15	8.2% (4.2%–12.3%)
	Muscle endurance functions	4	2.2% (0.0%–4.3%)
	**Total**	**19**	10.4% (6.0%–14.9%)
Functions of the skin	Functions of the skin	2	1.1% (–0.4%–2.6%)
	Functions of the hair and nails	1	0.5% (–0.5%–1.6%)
	**Total**	**3**	1.6% (–0.2%–3.5%)
Others	Functions related to the digestive system: Teeth	15	8.2% (4.2%–12.3%)
	Functions of the hematological and immunological systems: Anti-oxidants	8	4.4% (1.4%–7.4%)
	Growth	9	4.9% (1.8%–8.1%)
	**Total**	**32**	18.1% (12.5%–23.8%)
**TOTAL**		**185**	100.0% 5.2% (4.2%–6.2%)

For details see http://apps.who.int/classifications/icfbrowser/ [29].

## Supplementary Materials

The following are available online at http://www.mdpi.com/2072-6643/8/3/137/s1, Table S1: Prevalence of nutrition and health claims (including symbolic ones) by food category, Table S2: Food Categories used in the data collection according to Dunford *et al.*: International collaborative project to compare and monitor the nutritional composition of processed foods, *Eur. J. Prev. Cardiol.* **2012**, *19*, 1326–1332.
