# Supplementary material for: Prevalence of Nutrition and Health-Related Claims on Pre-Packaged Foods: A Five-Country Study in Europe
Source: Nutrients. 2016 Mar 3;8(3):137. doi: 10.3390/nu8030137 (PMC4808866; doi:10.3390/nu8030137)
Supplement: Supplementary file 1 [file nutrients-08-00137-s001.docx]

Supplementary Materials: Prevalence of Nutrition and Health-Related Claims on Pre-Packaged Foods: A Five-Country Study in Europe

Sophie Hieke, Nera Kuljanic, Igor Pravst, Krista Miklavec, Asha Kaur, Kerry A. Brown, Bernadette M. Egan, Katja Pfeifer, Azucena Gracia and Mike Rayner

**Table S1.** Prevalence of nutrition and health claims (including symbolic ones) by food category.

| **Food Group** | **No. of Foods** | **Nutrition Claims ^a^** | | | **Health Claims** | | | **...of Which Are Symbolic Claims** | | | **NO CLAIMS** | |
| --- | --- | --- | --- | --- | --- | --- | --- | --- | --- | --- | --- | --- |
|  |  | **Number of Nutrition Claims** | **No. of Foods with Nutrition Claims** | **% of Foods with Nutrition Claims (95% CIs), 1dp** | **Number of Health Claims** | **No. of Foods with Health Claims** | **% of Foods with Health Claims (95% CIs), 1dp** | **No of Symbolic Claims** | **No. of Foods with Symbolic Claims** | **% of Foods with Symbolic Claims (95% CIs), 1dp** | **Foods without Any Claims** | **%, (95% CIs), 1dp** |
| Beverages | 265 | 149 | 81 | 30.6%  (25.0%–36.1%) | 80 | 45 | 17.0%  (12.4%–21.5%) | 12 | 12 | 4.5%  (2.0%7.0%) | 159 | 60%  (54.1%–65.9%) |
| Bread & bakery foods | 176 | 58 | 32 | 18.2%  (12.4%–23.9%) | 5 | 5 | 2.8%  (0.4%–5.3%) | 1 | 1 | 0.6%  (−0.6%–1.7%) | 140 | 79.5%  (73.5%–85.6%) |
| Cereal and cereal foods | 147 | 117 | 46 | 31.3%  (23.7%–38.9%) | 45 | 24 | 16.3%  (10.3%–22.4%) | 9 | 8 | 5.4%  (1.7%–9.2%) | 92 | 62.6%  (54.7%–70.5%) |
| Confectionary | 178 | 52 | 26 | 14.6%  (9.4%–19.8%) | 28 | 14 | 7.9%  (3.9%–11.9%) | 5 | 4 | 2.2%  (0.0%–4.4%) | 147 | 82.6%  (77.0%–88.2%) |
| Convenience foods | 209 | 22 | 18 | 8.6%  (4.8%–12.4%) | 8 | 8 | 3.8%  (1.2%–6.5%) | 6 | 6 | 2.9%  (0.6%–5.2%) | 183 | 87.6%  (83.0%–92.1%) |
| Dairy | 268 | 174 | 75 | 28.0%  (22.6%–33.4%) | 52 | 36 | 13.4%  (9.3%–17.5%) | 14 | 14 | 5.2%  (2.5%–7.9%) | 182 | 67.9%  (62.3%–73.5%) |
| Edible oils and oil emulsions | 31 | 21 | 8 | 25.8%  (9.5%–42.1%) | 16 | 8 | 25.8%  (9.5%–42.1%) | 5 | 5 | 16.1%  (2.4%–29.8%) | 21 | 67.7%  (50.3%–85.2%) |
| Eggs | 4 | 0 | 0 | 0 | 0 | 0 | 0 | 0 | 0 | 0 | 4 | 100% |
| Fish and fish foods | 78 | 30 | 20 | 25.6%  (15.7%–35.5%) | 11 | 6 | 7.7%  (1.6%–13.7%) | 1 | 1 | 1.3%  (−1.3%–3.8%) | 56 | 71.8%  (61.6%–82.0%) |
| Foods for specific dietary use | 41 | 82 | 32 | 78.0%  (64.8%–91.3%) | 77 | 29 | 70.7%  (56.2%–85.3%) | 10 | 10 | 24.4%  (10.7%–38.1%) | 4 | 9.8%  (0.3%–19.2%) |
| Fruit and Vegetables | 209 | 64 | 33 | 15.8%  (10.8%–20.8%) | 29 | 21 | 10.0%  (5.9%–14.2%) | 7 | 7 | 3.3%  (0.9%–5.8%) | 156 | 74.6%  (68.7%–80.6%) |
| Meat and meat foods | 189 | 42 | 18 | 9.5%  (5.3%–13.7%) | 13 | 11 | 5.8%  (2.5%–9.2%) | 6 | 6 | 3.2%  (0.7%–5.7%) | 166 | 87.8%  (83.1%–92.5%) |
| Sauces and spreads | 146 | 22 | 14 | 9.6%  (4.8%–14.4%) | 14 | 9 | 6.2%  (2.2%–10.1%) | 5 | 5 | 3.4%  (0.4%–6.4%) | 123 | 84.2%  (78.3%–90.2%) |
| Snack foods | 68 | 17 | 14 | 20.6%  (10.7%–30.4%) | 8 | 2 | 2.9%  (−1.2%–7.1%) | 0 | 0 | 0 | 54 | 79.4%  (69.6%–89.3%) |
| Sugars, honey and related foods | 25 | 15 | 6 | 24.0%  (6.0%–42.0%) | 6 | 4 | 16.0%  (0.6%–31.4%) | 0 | 0 | 0 | 19 | 76%  (58.0%–94.0%) |
| **TOTAL** | **2034** | **865** | **423** | **20.8%  (19.0%–22.6%)** | **392** | **222** | **11.0%  (9.6%–12.3%)** | **81** | **79** | **3.8% (3.0%–4.7%)** | **1,506** | **74.0%  (72.1%–75.9%)** |

^a^ Number (and the corresponding %) of foods with at least one nutrition or health claim. A food can have one or more nutrition and/or health claims. Please note that ‘health-related ingredients claims’ are not included in this table.

**Table S2.** Food Categories used in the data collection according to Dunford *et al.*: International collaborative project to compare and monitor the nutritional composition of processed foods, *Eur. J.* *Prev.* *Cardiol*. **2012**, *19*, 1326–1332.

| **Food Group** | **Food Category** | **Description** |
| --- | --- | --- |
| **Beverages** | Fruit and vegetable juices | Fresh and ambient fruit and vegetable juices |
|  | Soft drinks | Sugar-sweetened and artificially-sweetened soft drinks |
|  | Cordials | Cordials |
|  | Coffee and tea | Coffee and tea products |
|  | Electrolyte drinks | Sports electrolyte drinks |
|  | Alcoholic beverages | All alcoholic beverages |
|  | Waters | Plain and flavoured waters |
| **Bread and bakery products** | Bread | White, wholemeal and mixed grain/seed sliced bread and rolls  Fruit bread and fruit-based muffins/rolls  Wraps and other flatbread products  Turkish pide, bagels, English-style muffins, crumpets, pizza bases and other plain bread-based products |
|  | Biscuits | Filled and unfilled sweet biscuits  Flavoured and plain crisp bread and crackers |
|  | Cakes, muffins & pastry | Scones, pikelets, doughnuts, cakes, sweet buns, pancakes, crepes, muffins (cake-style), slices *etc.*  Cake, pikelet and pancake dry mixes  Sweet pastries (fresh, ambient, chilled and frozen) |
| **Cereal and cereal products** | Cereal bars | Plain, chocolate-topped and yoghurt-topped cereal-based bars |
|  | Noodles | Flavoured and plain dry packet and fresh noodles |
|  | Breakfast cereals | Ready to eat breakfast cereals  Oats and other breakfast cereals that require heating  Other processed cereals (e.g., bran) |
|  | Pasta | Canned and ambient pasta and sauce (with and without meat) products (excludes frozen ready meals)  Packaged fresh pasta with sauce  Savoury/flavoured dry pasta-based side dishes  Plain dry pasta |
|  | Maize (corn) | Tortillas, tamales, tacos and other corn-based cereal products |
|  | Rice | Plain rice  Savoury rice-based side dishes |
|  | Couscous | Couscous side dishes and plain couscous |
|  | Unprocessed cereals | Flour and other unprocessed cereals (e.g., polenta, psyllium, bread crumbs, yeast) |
| **Confectionary** | Chocolate and sweets | Chocolate-based confectionery, sugar-based confectionery |
|  | Jelly | Jelly products and mixes |
|  | Chewing gum | All chewing gums and bubble gum products |
| **Convenience foods** | Pizza | Frozen and refrigerated pre-packed pizzas |
|  | Soup | Canned, chilled and ambient soup products |
|  | Ready meals | Frozen, chilled and ambient pre-prepared meals |
|  | Prepared salads and sandwiches | Chilled pre-prepared salads and sandwiches (excluding fast food) |
|  | Other | Other pre-prepared foods such as quiches and pasta |
| **Dairy** | Cheese | Feta, haloumi, parmesan and other high-salt cheeses  All types of full and reduced fat cheddar/Colby etc. cheese including shredded, block or sliced  Soft cheeses such as cream cheese, ricotta and cottage cheese  Processed cheese slices and products |
|  | Yoghurt products | Fruit, flavoured and natural yoghurts (full fat, reduced fat and skim varieties) including yoghurt drinks |
|  | Milk | Flavoured and unflavoured dairy milk products  Flavoured and unflavoured soymilks  Flavoured and unflavoured oat, almond and other milks Condensed, evaporated and powdered milk products (including coconut milk) |
|  | Cream | Thickened, sour and regular cream products |
|  | Deserts | Dairy-based desserts (e.g., custards, rice puddings)  Dairy-based dessert mixes (e.g., powders) |
|  | Ice cream and edible ices | Dairy and soy-based ice cream varieties and edible ices |

**Table S2.** *Cont.*

| **Food Group** | **Food Category** | **Description** |
| --- | --- | --- |
| **Edible oils and oil emulsions** | Butter and margarine | Salted and unsalted butter and margarine products |
|  | Cooking oils | Cooking oils such as olive oil, canola oil and other vegetable oils |
| **Eggs** | All egg products |  |
| **Fish and fish products** | Canned fish and seafood | Plain and flavoured canned tuna, salmon, sardines, anchovies, mackerel, herring, kipper, oysters and shellfish |
|  | Chilled fish | Chilled processed fish products (e.g., smoked salmon) |
|  | Frozen fish | Coated frozen fish products (e.g., fish fingers) and uncoated fish products |
| **Foods for specific dietary use** | Baby food | All infant formula products and baby food |
| **Fruit and vegetables** | Vegetables | Canned tomato products  Canned beans and peas  Baked beans in tomato sauce (with and without additions)  Canned creamed, plain and sweet corn  All other canned vegetables  Pickled vegetable and olive products  Frozen potato-based products  Frozen unprocessed vegetables |
|  | Fruit | Dried fruit products including coconut  Fruit-based bars  Fruit products canned in juice or syrup  Fruit gels, fruits in jelly and fruit puree |
|  | Jams and spreads | Jams, marmalades and other preserves |
|  | Nuts and seeds | Salted and unsalted nuts and seeds |
| **Meat and meat products** | Processed meat and derivatives | Pre-packed bacon products  Beef, pork, chicken and lamb sausages and chilled hot dogs  Pre-packaged sliced deli meats  Pre-packaged salami and cured meats  Beef, pork, chicken and lamb meat burgers  Canned meat products (excluding soup and pasta)  Frozen meat pies, sausage rolls and other meat-based pastry products such as dim sums |
|  | Meat alternatives | Plain tofu and other meat-free alternatives  Meat-free products (e.g., meat-free sausages) |
| **Snack foods** | Crisps and snacks | Plain and flavoured potato crisps  Plain and flavoured snack foods  Extruded snacks (e.g., cheesy snacks)  Plain and flavoured corn chips  Pretzels, popcorn and other snack foods  Other fried snack foods (e.g., plantain chips)  All varieties of cracker-based snack packs |

**Table S2.** *Cont.*

| **Food Group** | **Food Category** | **Description** |
| --- | --- | --- |
| **Sauces and spreads** | Sauces | Table sauces such as tomato sauces and ketchups, sweet chilli, BBQ sauces, Steak, HP and Worcestershire sauces  Soy, fish, oyster and other Asian high-salt sauces  Mustard products  Marinade products  Meat accompaniments (e.g., apple, cranberry and mint sauces)  Plain and flavoured tomato paste products  Asian and Indian flavoured powdered, ambient and liquid meal-based sauces  Ambient and fresh pasta sauces  Recipe bases  Liquid and powdered gravies and stock |
|  | Mayonnaise/Dressings | Full and low-fat mayonnaise  Oil-based, vinegar-based and other types of salad dressing |
|  | Spreads | Crunchy and smooth salted and unsalted peanut butter  Relishes, chutneys and pickles  Other savoury spreads (e.g., vegetable spreads)  Pâté spreads  Sweet spreads  Yeast-extract spreads (e.g., vegemite)  Chilled and ambient dips and salsa |
| **Sugars, honey and related products** | Honey and syrups | Honey and syrups  Dessert toppings |

The categories were designed to be applicable internationally and based on existing food databases.
